# Antigen 85B of *Mycobacterium tuberculosis*: From Immunodominant Antigen to Vaccine Candidate

**DOI:** 10.3390/vaccines14090811

**Published:** 2026-09-15

**Authors:** Vivek Chauhan, Gaytri Mahajan, Rakesh Kumar, Shrikanth S. Gadad, Subhash C. Chauhan, Chinnaswamy Jagannath, Subramanian Dhandayuthapani

**Affiliations:** 1 Division of Cancer Immunology and Microbiology, Department of Medicine and Oncology and South Texas Center of Excellence in Cancer Research, School of Medicine, The University of Texas Rio Grande Valley, Edinburg, TX 78541, USA; vivek.chauhan@utrgv.edu (V.C.); gaytri.mahajan@utrgv.edu (G.M.); rakesh.kumar@utrgv.edu (R.K.); shrikanth.gadad@utrgv.edu (S.S.G.); subhash.chauhan@utrgv.edu (S.C.C.); 2 Department of Pathology and Genomic Medicine, Houston Methodist Research Institute & Weill Cornell Medical College, Houston, TX 77030, USA; cjagannath@houstonmethodist.org

**Keywords:** *M. tuberculosis*, vaccine, antigen 85B, immune response, subunit, recombinant, BCG, mRNA vaccine, DNA vaccine

## Abstract

Tuberculosis (TB), caused by *Mycobacterium tuberculosis* (Mtb), continues to pose a significant global health challenge despite the existence of effective treatments. The only licensed TB vaccine, Bacillus Calmette–Guérin (BCG), offers inconsistent and generally limited protection against adult pulmonary TB. As a result, the pursuit of more effective TB vaccines has intensified over the past two decades, prompting the evaluation of several immunodominant Mtb antigens as vaccines. Among these, antigen 85B (Ag85B) has emerged as a promising candidate due to its critical roles in mycobacterial cell wall biosynthesis, host cell adhesion, and the induction of immune responses. Ag85B has been incorporated into diverse vaccine platforms, including subunit, recombinant, DNA, and mRNA-based vaccines. Evidence from preclinical and clinical studies indicates that Ag85B-containing vaccine formulations elicit robust cellular immune responses, and in many instances, enhance protection against TB. Collectively, these findings position Ag85B as a central component in the development of next-generation TB vaccines. This review examines the biological properties and immunological significance of Ag85B and provides an overview of recent advances in Ag85B-based vaccine strategies.

## 1. Introduction

Tuberculosis (TB) is one of the most widespread causes of infectious disease-related mortality in the world [1]. *Mycobacterium tuberculosis* (Mtb), the pathogen that causes TB, is an intracellular bacterium that primarily infects the lungs but can spread to other organs [2,3]. Bacillus Calmette–Guérin (BCG) is the only commercially approved vaccine against TB, which has been widely used for over a century [4,5]. Even though BCG can protect against severe forms of TB in children, its efficacy against pulmonary TB in adults is quite inconsistent and usually unsatisfactory [6]. This shortcoming has led to extensive research efforts to develop next-generation TB vaccines that can protect against TB effectively [7,8,9,10,11].

Studies have shown that activation of cell-mediated immune responses, specifically Th1-type CD4^+^ T cells, leads to the enhanced secretion of cytokines such as interferon-γ (IFN-γ), tumor necrosis factor-α (TNF-α), interleukin-2 (IL-2), and Th17 responses, resulting in IL-17 production, which is essential to provide protective immunity against Mtb [12,13,14,15]. These cytokines activate macrophages and enhance their capacity to eliminate intracellular Mtb [14,16]. Consequently, many TB vaccine development strategies aim to induce high-quality antigen-specific T-cell responses against key mycobacterial proteins [17,18,19,20,21,22]. Among all antigens studied, antigen 85B (Ag85B) has emerged as one of the most promising candidates due to its strong immunogenicity and its significance in the physiology of Mtb [10,23,24,25,26].

Ag85B, along with Ag85A and Ag85C, is part of the antigen 85 family, a group of secreted proteins involved in the biosynthesis of the mycobacterial cell wall [27,28]. These proteins act as mycolyltransferases, catalyzing the transfer of mycolic acids to cell wall components, thereby conferring structural stability for Mtb [28,29]. Since it is abundantly secreted during infection and is highly recognized by host immune cells, Ag85B has been widely investigated as a target antigen for TB vaccine development [18,20,30,31,32]. Several studies have shown that Ag85B can induce a robust T-cell response, making it an attractive candidate for a subunit and recombinant vaccine platform [7,8,17,20,33,34,35]. Although Ag85B of Mtb plays an important role in host–pathogen interactions [34] and is used as a diagnostic tool for TB [36,37], this review will highlight its significance in various anti-tuberculosis vaccines.

## 2. Biology of Ag85B and Its Role in Mtb

**Figure 1:**
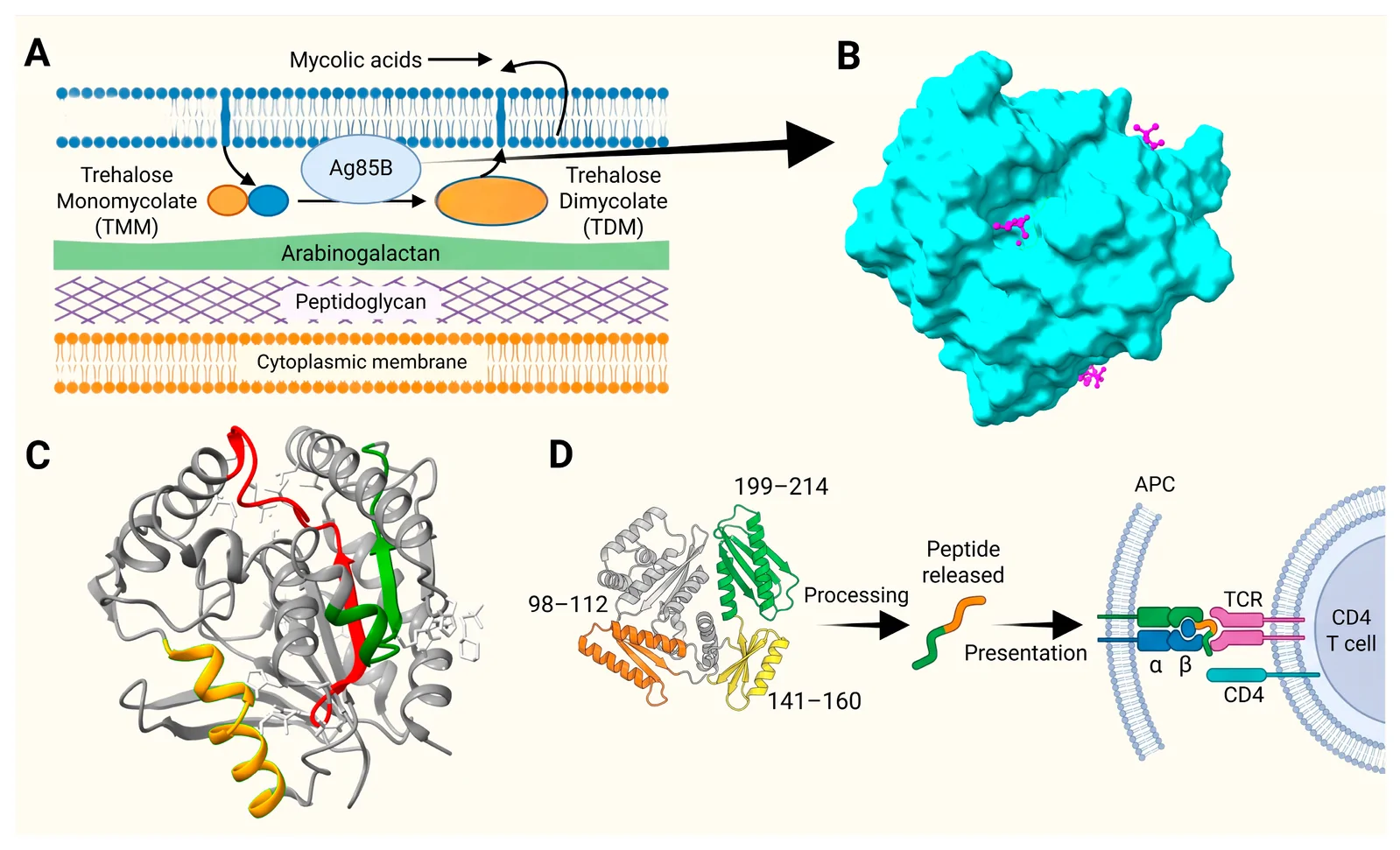
Structural and functional overview of Ag85B. (**A**) Schematic presentation of Ag85B localized within the Mtb cell membrane, denoting its role in the catalytic transfer of mycolic acid from TMM to TDM during cell wall biosynthesis. (**B**) 3D surface model of Ag85B (PDB ID: 1F0N), denoting the structural topology of the protein. The catalytic residues associated with the mycolyltransferase active site are highlighted in magenta. The surface depiction emphasizes the spatial organization of proteins and residues contributing to substrate recognition and lipid transfer activity. (**C**) Immunogenic and antigenic regions mapped onto the protein structure. The selected regions (residues 96–112, 141–160, and 199–214), highlighted with orange, red, and green, respectively, illustrate the spatial distribution of experimentally characterized T-cell response regions of Ag85B [66,67,68]. (**D**) Overview of Ag85B-mediated immune response: after processing, the peptides derived from Ag85B are loaded onto the MHC class II (αβ) molecule for antigen presentation, which are then presented to CD4^+^ T cells, leading to cytokine production and the activation of adaptive immunity. Created in BioRender. Dhandayuthapani, S. https://BioRender.com/jixqvhf accessed on 11 September 2026.

Three closely related secreted proteins, Ag85A (31 kDa; Rv3804c/fbpA), Ag85B (30 kDa; Rv1886c/fbpB), and Ag85C (31.5 kDa; Rv0129c/fbpC), encoded by the fibronectin (*fbp*) gene family, comprise the antigen 85 complex in Mtb [38,39,40]. They function as mycolyltransferases and catalyze the transfer of mycolic acid during the assembly of complex structures in the cell wall biosynthesis of Mtb [27,41]. In addition, the Ag85 complex functions as a fibronectin-binding protein, which facilitates the interaction of Mtb with host tissues, resulting in bacterial colonization and pathogenesis [39,42]. Among these three, Ag85B is the most abundantly secreted protein during the bacterial growth phase and is omnipresent across the cell wall [3]. The mycobacterial cell wall is rich in lipids, which help mycobacteria survive harsh environmental conditions and resist antibiotics as well as the host immune response [3,27]. Ag85B enzymatic activity is vital for maintaining the robust structural integrity of the mycobacterial cell wall. High levels of long-chain mycolic acids in mycobacterial cell walls (Figure 1A) limit the penetration of antimicrobial compounds into the cell, thereby enhancing bacterial survival in host macrophages [43,44]. Ag85B architecture reveals a conserved α/β fold characteristic of the mycolyltransferase family and associated with substrate binding and enzymatic activity (Figure 1B) [28,45]. Ag85B also mediates the synthesis of trehalose dimycolate (TDM), which is a major cell wall glycolipid (Figure 1A) [46]. TDM, also known as cord factor, is not only a key structural component of the bacterial cell wall, but also an important virulence-associated molecule [47]. Therefore, Ag85B affects both the virulence and structural stability of Mtb.

High expression level and extracellular localization of Ag85B make it a potent candidate for uptake and presentation via host antigen-presenting cells (APCs), including macrophages and dendritic cells. Early studies showed that efficient Ag85B processing in macrophages depends on phagosomal acidification via the activation of vacuolar cathepsin D and recruitment of ATPases [48]. Ag85B contains specific immunogenic regions that serve as important binding sites for T-cell recognition (Figure 1C) [45]. After uptake in the cell, Ag85B is degraded in the endosomal-lysosomal pathway, followed by peptide presentation on major histocompatibility complex II (MHC-II) molecules to the CD4^+^ T lymphocytes (Figure 1D) [49]. Ag85B is known to have several well-characterized epitopes that contribute to its potent immunogenic profile in both humans and mice [50,51]. Amongst these, the P25 epitope (FQDAYNAAGGHNAVF) is widely studied and is known to induce MHC-II-mediated immunity incorporating robust IFN-γ production [50,52]. Mainly, Ag85B induces a T-helper 1 (Th1)-mediated immune response, which results in the activation of CD4^+^ T-cells, leading to the enhanced production of cytokines such as tumor necrosis factor-α (TNF-α), interferon-γ (IFN-γ), and interleukin-2 (IL-2), which play an important role in protective immunity against TB [53,54,55]. IFN-γ serves as a master regulator of macrophage activation, increasing their bactericidal capacity by enhancing the production of reactive oxygen species (ROS) and promoting phagosome–lysosome (PL) fusion [56,57,58]. TNF-α plays a pivotal role in enhancing granuloma structural integrity, effectively surrounding bacteria and preventing their spread throughout the body [16,59]. The synchronized production of all of these cytokines enhances cell-mediated immunity, which is key to regulating Mtb infection within the host [16,60,61]. In addition, Ag85B is also a strong target for humoral immunity [62,63]. Ag85B-specific IgG antibodies have been detected in TB patients and are being explored as potential biomarkers against TB [36,62]. Furthermore, vaccine formulations containing Ag85B steadily elicit a strong antibody-mediated immune response coupled with T-cell-mediated immunity [64,65]. This dual nature of Ag85B in inducing a strong immune response in the host and its role in cell wall biosynthesis in Mtb makes it an attractive target for TB vaccine development.

## 3. Vaccines Based on Ag85B

**Figure 2:**
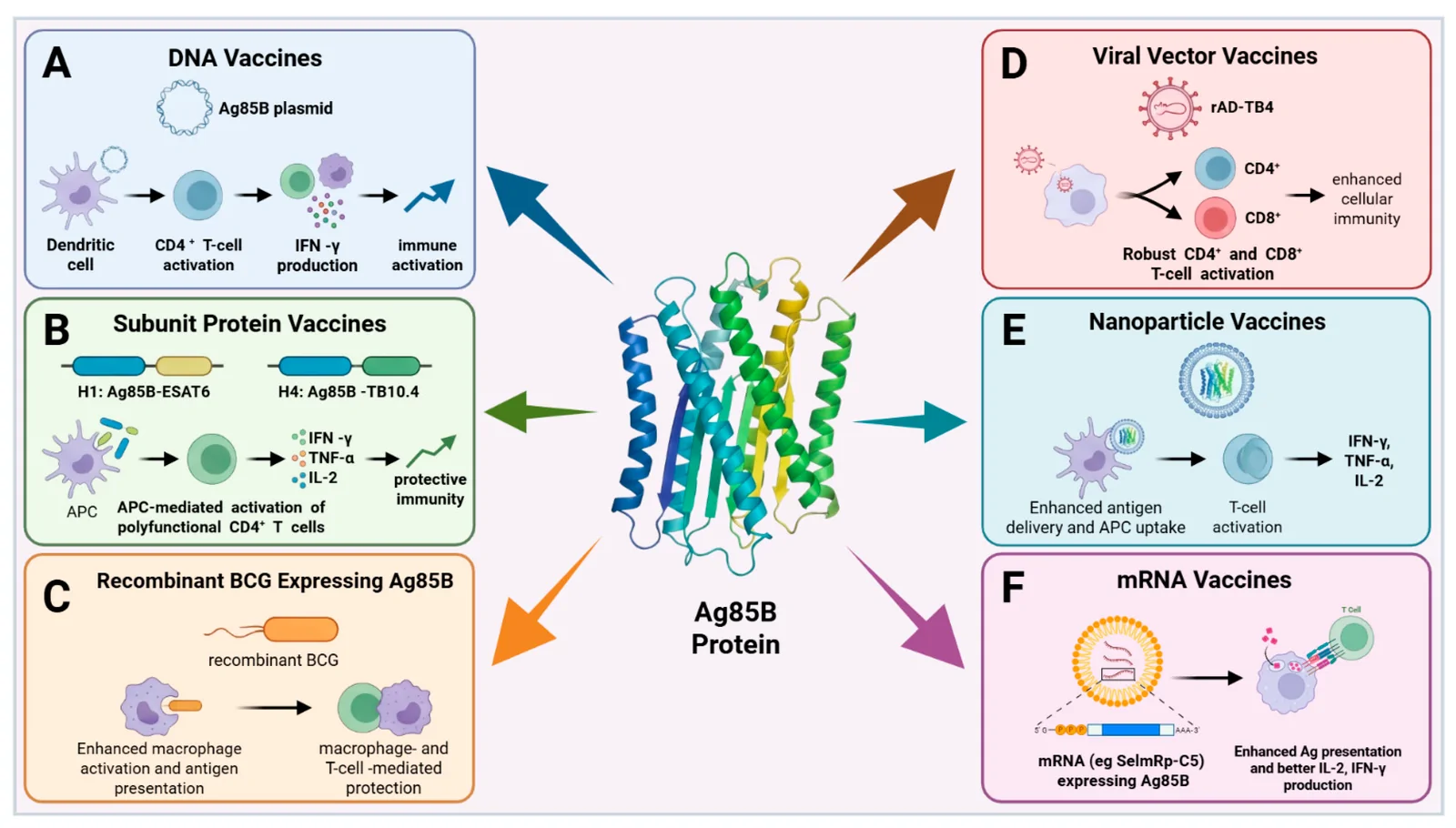
Ag85B-based vaccine strategies against TB. The illustration aims to depict different approaches used for Ag85B (the central element in the figure) in TB vaccine development. (**A**) DNA vaccine utilizes an Ag85B plasmid that has induced an enhanced T-cell response in preclinical models [25]. (**B**) Subunit vaccines like H1 and H4 combine Ag85B with other antigens to induce a polyfunctional T-cell response and improved immunogenicity [11,93]. (**C**) Recombinant BCG/Mycobacterial strains engineered to overexpress Ag85B show better protection than conventional BCG [33]. (**D**) Viral vector vaccines, such as rAD-TB4, use adenovirus for Ag85B delivery, resulting in robust CD4^+^ and CD8^+^ T-cell responses in clinical studies [87]. (**E**) NP-based delivery systems improve Ag85B stability and enhance targeted delivery, resulting in increased overall immunity [94]. (**F**) mRNA vaccines as BCG booster (SelmRp-C5) encode Ag85B to enhance protective T-cell immunity and reduce bacterial load [95]. Collectively, these approaches show the diverse nature of Ag85B as a key component for next-gen TB vaccine development. Created in BioRender. Dhandayuthapani, S. https://BioRender.com/1f003xy accessed on 11 September 2026.

Ag85B is among the most extensively studied Mtb antigens due to its high abundance during bacterial replication, robust secretion, and ability to elicit strong CD4^+^ T-cell-mediated immune responses. Since the initiation of Mtb vaccine development in the early 2000s, multiple strategies have been implemented to enhance the immunogenicity and delivery of Ag85B (Table 1, Figure 2) [34,64,69]. The following section summarizes the Ag85B-based vaccine strategies, organized by platform and developmental approach.

### 3.1. Subunit Vaccines

Subunit vaccines based on recombinant proteins represent a conventional yet continuously evolving strategy for TB vaccine development, offering advantages such as enhanced safety, well-defined formulations, and flexibility in antigen selection. In a bid to improve the efficacy of the vaccine, scientists have increasingly employed antigen fusion strategies whereby Ag85B is combined with other immunodominant mycobacterial antigens [20,35,64,70,71]. The rationale behind these fusion constructs is to enhance immune responses and broaden antigenic coverage by incorporating antigens expressed at different stages of the bacterial life cycle. A well-studied example is the Ag85B–ESAT-6 fusion protein, which has been extensively evaluated and shown to enhance immunogenicity and induce long-lasting T-cell responses in mouse models [34,72,73,74]. A clinical study further revealed that vaccination with Ag85B–ESAT-6 formulated with the IC31 adjuvant can elicit strong antigen-specific cellular immune responses that persist for extended periods after immunization in naïve human volunteers [11]. The subsequent work on this approach led to the development of multistage vaccines like H56, which combined both Ag85B and ESAT-6 with a latency-associated antigen, Rv2660c [64,75,76].

A study reported the in silico design of a multi-epitope ESAT-6:Ag85B: Fcγ2a fusion protein as a novel candidate for a TB vaccine, representing a computational approach to optimizing vaccine design [77]. Here, Fcγ2a was integrated as a targeted delivery system for two secretory proteins. This novel multi-epitope was assessed by numerous online tools as an optimal TB vaccine; however, its expression, immunogenicity, safety, and protective efficacy remain experimentally unvalidated. Another study showed that fusing Rv2299c, a dendritic cell-activating protein, enhanced the protective immunity conferred by the Ag85B–ESAT6 vaccine against TB in a mouse model [78]. This recombinant Rv2299c–Ag85B–ESAT6 protein facilitated rapid dendritic cell (DC) activation and maturation, resulting in a significantly higher production of TNF-α and IL-2 in the lungs, spleens, and lymph nodes of immunized mice compared with those with Ag85B–ESAT6 alone. Similarly, development and initial assessment of a vaccine candidate comprising HspX, CFP-10, Ag85B, and ESAT-6 led to notably enhanced Ag-specific IFN-γ responses, higher IL-12 secretion, and increased IgG production in immunized mice when compared to BCG, highlighting the importance of adjuvant-associated stimulation of both humoral and cellular immunity [79]. A vaccine candidate containing Ag85B fused to the Fc fragment of human IgG1 also resulted in improved antigen uptake by APCs via Fcγ receptors, leading to higher IFN-γ production and an enhanced Th-1-biased immune response in immunized mice [80].

In addition, Ouyang et al. (2025) [31] demonstrated that the Ag85B–Rv2660c–MPT70 fusion protein (ARM) increased BCG-induced immunity by enhancing antigen-specific B and T cell responses, reducing Mtb burden in mice’s lungs, and boosting Th-1-type multifunctional CD4^+^ T cell responses. A parallel study showed that the Rv2299cD2D3–ESAT-6–Ag85B fusion protein (REA) induced DC maturation and Th1/Th17 responses, resulting in enhanced maturity and reduced Mtb load in the mouse model [81]. These findings indicate that multistage vaccines comprised of both actively replicating and dormant bacterial antigens can be more effective than single-antigen vaccines. Together, these studies suggest that Ag85B, when fused with other immunogens, leads to enhanced antigen recognition and presentation, stronger immunity, and a notable decrease in mycobacterial burden in test subjects.

### 3.2. Recombinant BCG Vaccines Overexpressing Ag85B

Acknowledging that BCG, though safe and widely adopted, has limited efficacy, many scientists have engineered a recombinant BCG (rBCG) strain that overexpresses Ag85B and other immunogenic antigens to enhance vaccine efficacy while maintaining its safety profile. Pioneer work was conducted by Horwitz et al. (2000) [69], who demonstrated that an rBCG strain engineered to overexpress Ag85B induced a robust antigen-specific immune response and provided immune protection against Mtb in mice. Building on this foundation, Jagannath et al. (2009) demonstrated that the rBCG overexpressing Ag85B induces autophagy in APCs, resulting in stronger immunogenicity in mice, implying that vaccine efficacy can be increased by boosting autophagy-mediated antigen presentation [82]. Subsequent studies showed that pharmacologically or genetically enhanced lysosomal trafficking or autophagy via rBCG enhanced Ag85B epitope presentation and significantly augmented immunogenic efficacy against Mtb. This emphasized the role of Ag85B as the central immunodominant antigen whose immunogenicity can be markedly increased via modulation of antigen processing pathways [83]. Wang et al. (2012) [84] developed a BCG strain engineered to overexpress both Ag85A and Ag85B. This strain exhibited enhanced immunogenicity and protective efficacy, as evidenced by increased IFN-γ production and reduced bacterial burden in the mouse model [84]. More advanced versions of the rBCG construct have been engineered to overexpress different immunogenic antigens alongside Ag85B. For instance, rBCG::XB, a rBCG strain overexpressing Ag85B and HspX, provided stronger immunogenicity against both active and latent phases of Mtb infection [85]. Cumulatively, these studies provide compelling evidence supporting the potential of Ag85B as a critical antigen for the development of effective TB vaccines.

### 3.3. Viral Vectored Vaccines

Viral vector-based vaccines represent another important platform for not only delivering antigen, but also harnessing the capacity of an attenuated virus to elicit a robust immune response within the host [86]. For this purpose, adenoviral vectors and modified vaccinia virus Ankara (MVA) have gained attention for their ability to transduce a diverse range of cell types and stimulate robust polyfunctional T cell responses. An adenovirus (Had5 viral platform)-based vaccine named rAD-TB4, containing multiple antigens such as Ag85B–ESAT6–Mpt64–Rv2660c, showed enhanced antigen-specific cellular responses, reduced pulmonary infection, and decreased bacterial load following Mtb infection when compared to BCG in 17-week-old mice [87]. Similarly, a vaccine candidate was developed using a recombinant vaccinia virus overexpressing Ag85B, which demonstrated stronger immunity against Mtb [9].

A concurrent study used a heterologous viral vector setup (different viral vectors for priming and boosting) to express Ag85B–TB10.4. This vaccine generated significantly higher levels of IFN-γ-producing splenocytes, CD69^+^CD8^+^ T cells, IFN-γ secretion, and antigen-specific antibodies compared with BCG in a mouse model [88]. Simultaneously, clinical evaluation has commenced for viral vector vaccines. MVA85A is a modified vaccinia Ankara virus expressing the antigen 85A vaccine designed to enhance the protective efficacy of BCG. In several clinical trials, it has been shown to enhance BCG immunogenicity against TB [89,90,91]. Interestingly, Khan et al. developed an intranasal bovine adenovirus vaccine expressing the Ag85B-p25 epitope, which protected mice against Mtb as both a BCG booster and a primary vaccine [52,83,92].

### 3.4. DNA Vaccines

DNA vaccines are considered one of the first and most direct strategies for developing Ag85B-based immunotherapies. The mode of action of these vaccines is via the delivery of DNA encoding Ag85B (DNA-85B), enabling host cells to produce antigen within the cell and trigger both humoral and cellular immune responses. Pioneering work by Palma et al. (2007) [96] demonstrated that mice vaccinated with DNA-85B showed greater protection against Mtb in a mouse model. This protection was associated with CD4^+^ T cells producing interferon-gamma (IFN-γ) and restricting Mtb growth [96]. Following this initial observation, subsequent preclinical studies highlighted the significant protective and therapeutic potential of Ag85B vaccines [97,98]. Researchers found that the chimeric Ag85B/A DNA vaccine showed therapeutic and immunogenic effects on mice against TB, including multidrug-resistant TB [97]. Furthermore, the therapeutic potential of the DNA vaccine was exhibited through a multi-antigen approach. A B21 DNA vaccine expressing Ag85B, Rv1738, and Rv2029c conferred a strong therapeutic effect against latent Mtb infection, with an augmented Th1/Th17 and CD8^+^ cytotoxic T lymphocyte immune response and enhanced development of memory T cells in a preclinical model [99]. These studies provide an important mechanistic insight into how DNA vaccines encoding multifunctional fusion antigens can optimize protective immunity against TB.

Interestingly, Ag85B has also been examined as an immunological adjuvant beyond its conventional role as a vaccine agent [100,101]. Takamura et al. highlighted that the inclusion of Ag85B in an HIV gp120 DNA vaccine increases gp120-specific Th1 and cytotoxic T-cell responses, indicating that Ag85B can elicit immunomodulatory effects when co-administered with an unrelated antigen. This adjuvant effect was further enhanced in BCG-primed mice, where Ag85B + gp120 showed a substantially elevated cellular immune response [101].

### 3.5. mRNA Vaccines

COVID-19 gave a huge boost to the emergence of mRNA-based vaccines, which in turn opened new opportunities for the development of a TB vaccine. mRNA vaccines offer notable advantages over conventional vaccines, including rapid production, enhanced biosafety due to non-integration into the host genome, and the ability to deliver multiple antigens in a single formulation, such as lipid nanoparticles (LNPs). Ag85B is a preferred antigen for mRNA vaccine research against TB. In another study, mRNA encoding Ag85B formulated in LNPs was utilized as a prime-booster for BCG-immunized mice, which enhanced the immune responses in mice, revealing that it can be used both as an independent vaccine and as a BCG booster dose [55]. Another study engineered a novel self-adjuvanted mRNA vaccine, SelmRp-C5, which expressed the Ag85B-p25 epitope, elicited robust antigen presentation to T-cells in mouse dendritic cells and macrophages, as well as in human macrophages, and increased cytokine production. This vaccine helped in a 1 log10 decrease in Mtb load and enhanced IFN-γ and IL-2 secretion when compared to BCG [95].

The emergence of multiantigen mRNA vaccines further broadened the potential of this platform. An mRNA vaccine recently developed (QTAP-R), containing multiple antigens, Ag85B, Hsp70, and ESAT-6, enhanced both cellular and humoral immune responses in mice immunized with two doses [102]. Notably, Vidal et al. [11] identified Ag85B as one of the eight most protective Mtb antigens and added it to a tetravalent mRNA-LNP vaccine formulation along with Rv0287, Rv1387, and Rv1788, indicating the potential of Ag85B as a component of a multivalent mRNA-based TB vaccine [103]. Another vaccine, mRNA^CV2^, encoding a CysD/Ag85B fusion protein, has been shown to enhance Th1 CD4^+^ T-cell responses in the blood and lungs and confer increased protection against Mtb [104]. Furthermore, an mRNA vaccine that fuses Mtb epitopes, including Ag85B, was also developed [7]. This vaccine enhanced Th1 responses in mouse models by increasing the production of key cytokines, including IFN-γ and IL-2, highlighting Ag85B as a promising antigen for the development of effective mRNA vaccines against TB.

**Table 1:** Recent studies on Ag85B-based vaccine development.

S. No.	Platform	AG85B Use	Experimental Model/Level of Evidence	Key Findings and Evidence of Protection	Reference
1.	Recombinant bacterial vaccine	Mycobacterium smegmatis expressing Ag85B	Mtb-infected mice/Preclinical	Enhanced humoral and cellular immunity and promoted Th1/Th2 cytokine response. Reduced bacterial load in spleen.	[33]
2.	Recombinant bacterial vaccine/DNA booster	rBCG-Mkan85B and DNA-Mkan85B	Mice/preclinical	Enhanced Ag85B-specific CD8^+^ T-cell responses. Showed protection against *M. kansasii* with reduced pulmonary bacterial burden.	[105]
3.	DNA vaccine (W545)	Multiepitope DNA vaccine including Ag85B epitopes	Mouse/Preclinical	Triggered Th1 immune responses and reduced lung pathology.	[106]
4.	mRNA vaccine	Circular mRNA containing Ag85B epitopes	Computational/In silico	Predicted strong Th1 responses, enhanced IL-2 and IFN-γ production, and durable immune memory. Although the vaccine candidate has not been evaluated experimentally.	[7]
5.	BCG/mRNA-LNP booster	Ag85B mRNA booster after BCG priming	Mice/preclinical	Produced stronger T-cell responses and improved humoral response. Functional inhibition demonstrated; protection was not the endpoint.	[55]
6.	Subunit protein vaccine	Ag85B-Rv2660c-MPT70 fusion protein	H37Ra infected mice/Preclinical	Induced strong CD4^+^ T-cell and antibody responses following BCG vaccination. Reduced bacterial burden and pathology in lungs.	[35]
7.	Subunit protein vaccine	Ag85B fused with human Fcy1	Mice/preclinical	Improved antigen presentation and enhanced IFN-y production. Protection not evaluated.	[80]
8.	Subunit protein vaccine	CFP10-Ag85B fusion protein with cholera toxin non-toxic B subunit (CTB)	Wistar rats/other animal model	Demonstrated higher antigenicity in silico and induced strong antigen-specific IgG responses following immunization. Protection was not established by Mtb challenge.	[71]
9.	Viral vector vaccine	Recombinant vaccinia virus expressing Ag85B	H37Rv and HN878 challenged mice/preclinical	Reduced lung bacterial load in prime-boost vaccination mode. Reduced lung bacterial burden after H37Rv and hn878 challenge.	[9]
10.	Subunit vaccine with adjuvant	Ag85B with TLR4 adjuvant and STING agonist	Mtb-challenged mice/preclinical	c-di-GMP enhanced Ag85B based Th1-biased CD4^+^ T-cell responses. Reduced bacterial burden after Mtb challenge.	[107]
11.	Microneedle patch vaccine	Inactivated *M. paragordonae* preserving Ag85B antigen	BCG-primed mice and H37Rv challenged/preclinical	Enhanced BCG-induced protective immunity.	[108]
12.	Nanoparticle vaccine	PLGA nanoparticles encapsulating H1 (Ag85B–ESAT6)	Mtb-challenged mice/preclinical	A single dose initiates protective immunity against TB.	[109]
13.	Nanoparticle vaccine	PLA nanosponges encapsulating Ag85B (aPNS)	BCG-primed and Mtb-challenged mice/preclinical	aPNS were nontoxic, improved antigen stability, macrophage uptake, and protective immunity. Significantly reduced bacterial burden in lung and spleen.	[110]

### 3.6. Clinical Development of AG85B-Based Therapeutics

Over the past decade, several clinical and translational studies have assessed vaccines containing Ag85B in both preclinical models and human clinical trials to examine its safety and efficacy [7,8,80,94,111,112]. Many subunit vaccine formulations have been made with Ag85B combined with immunodominant antigens, such as ESAT-6 and TB10.4. A well-known example is H4 (Ag85B-TB10.4) [93]. These preparations are mostly delivered with potent adjuvants, such as CAF01 and IC31, which further enhance antigen presentation [11,113,114]. Clinical studies evaluating these vaccines have regularly shown favorable safety profiles and the capacity to induce antigen-specific cellular immune responses [11,111,112,115]. AERAS-402 is a good example, which expresses Ag85A, Ag85B, and TB10.4 and has been evaluated in multiple clinical trials, including infants and adults, alongside individuals with HIV infection [115,116]. A summary of the clinical studies of Ag85B vaccine candidates is presented in Table 2.

The first known clinical trial of the Ag85B vaccine candidate was conducted using a recombinant BCG strain that overexpressed Ag85B and showed enhanced production of both CD4^+^ and CD8^+^ cells in humans, with increased production of IFN-gamma [111]. Although most vaccine candidates are in phase I trials, some vaccines, such as AERAS-402 [115] and H1/IC31 [117], have entered phase II trials, highlighting the promising nature of these vaccine candidates, the result of over a decade of cumulative effort. H56:IC31 is by far the most advanced clinical development for an Ag85B-based tuberculosis vaccine. It combines Ag85B and ESAT-6 (6-kDa early secretory antigenic target) with the latency-associated antigen Rv2660c in the IC31 adjuvant. Developed to function as a multistage vaccine targeting both early and late phases of Mtb infection, H56:IC31 was designed to combine antigens expressed at different stages of Mtb infection. Several clinical trials have validated its immunogenicity and acceptable safety profile in both infected and uninfected adults, showing the initiation of antigen-specific IgG and Th1-CD4^+^ cell responses without serious adverse effects [76,113,115,118]. Recent clinical studies have further evaluated H56:IC31 in post-treatment TB patients; however, in spite of the strong immunogenicity and robust safety profile, the phase IIb trial did not establish protection against recurrent TB [118,119,120].

H4:IC31 is another clinically evaluated Ag85B-containing vaccine candidate developed as a booster vaccine for individuals previously vaccinated with BCG. It contains a combination of Ag85B and TB10.4 in the adjuvant IC31. Early phase I studies in BCG-vaccinated adults demonstrated that H4 was well-tolerated and induced persistent Ag85B- and TB10.4-specific Th1-type CD4^+^ T-cell responses, including IL-2, TNF-α, and IFN-γ production [93,112]. However, in a phase II clinical trial involving 990 BCG-vaccinated adolescents in South Africa, H4:IC31 did not meet the primary efficacy (9.4%) endpoint for preventing initial Mtb infection as defined by QuantiFERON-TB Gold (QFT) conversion [121]. A greater reduction was observed for sustained QFT conversion (secondary endpoint), but this finding provided only a modest efficacy signal and did not demonstrate clear protection. Although the vaccine showed a reduction in sustained QFT conversion (35% reduction), it was still statistically insignificant (*p* = 0.16) [121]. Overall, it suggests that strong vaccine-induced cellular immunogenicity and a reasonable safety profile do not inherently translate to significant protection against Mtb.

Evaluation of these studies shows some consistent themes. Firstly, Ag85B-incorporated vaccines are safe and capable of producing a strong antigen-specific cellular immune response, in which polyfunctional Th1-type CD4^+^ T cells mediate the immune response, accompanied by cell-mediated immune responses and the production of TNF-α, IFN-γ, and IL-2 [113,116,117]. Secondly, in almost all cases, combining Ag85B with other immunogenic antigens such as TB10.4 or ESAT-6 resulted in enhanced immune responses in clinical trials [11,113,116]. This extensive clinical evaluation of Ag85B-based vaccines underscores its enduring significance as a central antigen in the development of next-generation TB vaccines.

### 3.7. Computational Approach for Vaccine Development

The use of modern computational approaches and techniques, such as proteomics and bioinformatics, is further increasing the efficacy of Ag85B-based drugs. In a recent study, scientists optimized the structure of Ag85B using the ThermoMPNN protein design algorithm, resulting in a point-mutant protein that when formulated in a liposomal vaccine system, induced a heightened humoral immune response in mice. These mice showed a reduction in pulmonary bacterial load compared with wild-type mice upon aerosol challenge [94]. Another study used a computational approach to design circular mRNA-based vaccine candidates that contained multiple antigenic epitopes [7]. Bioinformatics tools were used to predict and analyze the W545 DNA vaccine, developed by integrating Ag85A and Rv1419 with epitopes from Ag85B, Rv3047, and Rv2628 [106]. This vaccine elicited an enhanced immune response compared with the wild-type vaccine. Overall, these studies highlight the potential of modern algorithms and computational techniques in advancing vaccine development. Collectively, all the recent preclinical developments (Table 1) highlight the versatility of Ag85B as an effective vaccine candidate, paving the way for a next-gen TB vaccine.

### 3.8. Advanced Delivery Systems: Lipid Nanoparticles and Liposomal Formulations

**Figure 3:**
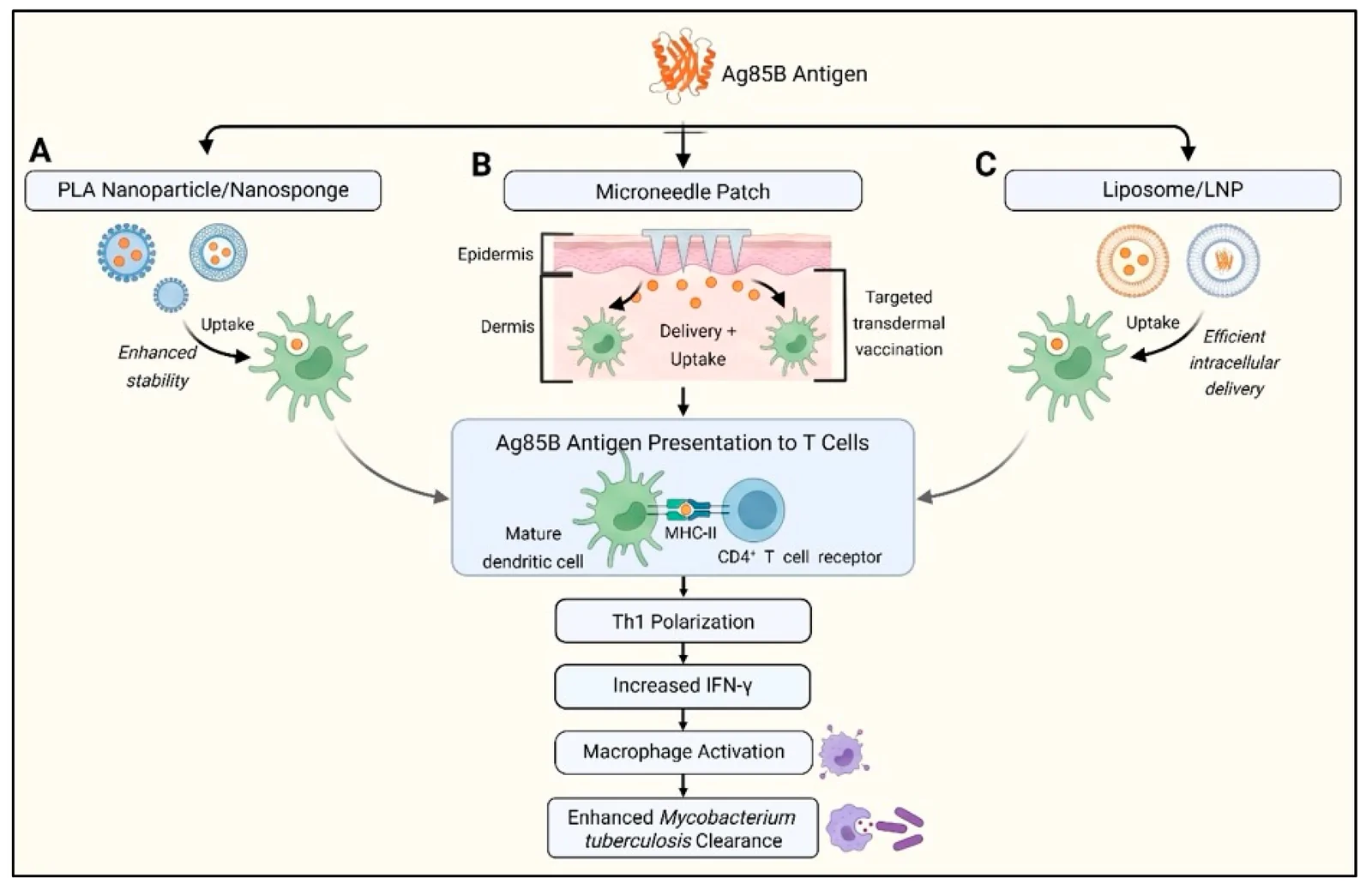
Schematics of novel antigen delivery strategies for Ag85B vaccines. The figure demonstrates advanced strategies to enhance the delivery and immunogenicity of Ag85B. Top: Ag85B antigen is depicted as the central protein target. Three distinct delivery strategies are shown: (**A**) Nanosponges/PLA nanoparticles, which encapsulate Ag85B and enhance the stability and uptake by dendritic cells, (**B**) microneedle skin patches, delivering Ag85B directly to dermal dendritic cells for targeted immune activation, and (**C**) liposomes/lipid nanoparticles (LNPs), facilitating improved cellular uptake and antigen presentation. Bottom: Arrows from all three strategies converge into dendritic cells presenting Ag85B to T cells, leading to downstream immune responses including Th1 polarization, IFN-γ production, macrophage activation, and enhanced bacterial clearance. Created in BioRender. Dhandayuthapani, S. https://BioRender.com/e8etxj7 accessed on 11 September 2026.

Advancements in drug delivery technologies have further expanded the potential of Ag85B vaccines. This advancement has been shaped by the recognition that the composition and the delivery route can significantly impact vaccine immunogenicity and protective efficacy. As a result, new platforms are being explored to enable more efficient and targeted vaccine delivery (Figure 3). Nanoparticle (NP)-based carriers, such as temperature-dependent polylactic acid (PLA) nanosponges, are being developed to enhance antigen stability and cell uptake, and to ensure a constant release. For instance, a new mRNA vaccine candidate containing Ag85B, encapsulated within a lipid nanoparticle, has shown promising results. When this vaccine was administered in combination with BCG, it showed enhanced polyfunctional T-cell response and improved inhibition of mycobacterial growth [55]. Another study showed that PLA-nanosponges loaded with Ag85B improved macrophage uptake and enhanced protective efficacy compared with BCG in a mouse model [110]. Diogo et al. (2019) [122] demonstrated that phosphatidylserine liposomes encapsulating Ag85B and ESAT-6 (Lipo-AE) were stable during storage and were readily taken up by Ag-presenting cells after being processed in the endoplasmic reticulum. Lipo-AE was able to induce a higher Th1/Th17-Th2 response to Ag85B and a reduced bacterial load in the lungs and spleen of infected mice [122]. Likewise, a microneedle patch-based booster dose containing *Mycobacterium paragordonae* containing Ag85B considerably increased Th1 immune responses [108]. These studies show that novel delivery strategies can further enhance vaccine efficacy.

**Table 2:** Translational and clinical studies investigating Ag85B-based vaccine candidates.

S. No.	Vaccine Candidate	Vaccine Platform	Study Phase	Population	Primary Endpoint	Key Reports	Reference
1.	H1 (Ag85B-ESAT-6)	Subunit vaccine with IC31 Adjuvant	Phase 1 clinical trial	Healthy Adults	Safety and immunogenicity study	Enhanced antigen-specific CD4^+^ T cell response	[11]
2.	H4 (Ag85B + TB10.4)	Subunit vaccine with IC31 Adjuvant	Phase I clinical trial	BCG-vaccinated South African adults	Safety and immunogenicity study	Acceptable safety in South African adults. Persistent polyfunctional T-cell responses	[93]
3.	H56 (Ag85B-ESAT-6 + Rv2660c)	Subunit vaccine with IC31 Adjuvant	Phase I clinical trial	Mtb infected/uninfected adults	Safety and immunogenicity study	Induced a strong CD4^+^ T cell response	[113]
4.	H1 + CAF01	Subunit vaccine with CAF01 adjuvant	Phase I Clinical trial	Healthy volunteers	Safety and immunogenicity study	Long-lasting T cell response (over 150 weeks)	[114]
5.	H1/IC31	Subunit vaccine with IC31 Adjuvant	Phase II, Double blind randomized trial	Mtb infected/uninfected adolescents	Safety and immunogenicity study	Safe for administration in HIV infected individuals. Induced long-lived TNF-α^+^IL-2^+^ CD4 responses	[117]
6.	H1/IC31 (R)	Subunit vaccine with IC31 Adjuvant	Phase II trial	HIV-infected adults, CD4 greater than 350 cells/mm^3^	Safety and immunogenicity study	Safe for administration in adolescents (uninfected and Mtb-infected), induced Ag85 B-specific CD4^+^ T cell response	[123]
7.	H4/IC31	Subunit vaccine with IC31 Adjuvant	Phase I Dose Escalation Trial	BCG-vaccinated adults	Safety and immunogenicity study	Dose combinations of 50 μg of H4 and 500 nmol of IC31 were found to be optimal for enhancing the immune response	[112]
8.	H4/IC31	Subunit vaccine with IC31 Adjuvant	Phase II	990 BCG-vaccinated, HIV-negative adolescents in South Africa	Safety and immunogenicity studies	Vaccines failed in preventing initial QFT conversion. Sustained QFT conversion at lower limits but not enough to reach statistical significance.	[121]
9.	H56/IC31	Subunit vaccine with IC31 Adjuvant	Phase I clinical trial	Adults treated for drug-susceptible pulmonary TB	Safety and immunogenicity study	Demonstrated durable Ag85B-specific immune response	[118]
10.	H56/IC31	Subunit vaccine with IC31 Adjuvant	Phase IIb clinical trial	HIV negative patients with completed treatment for drug susceptible pulmonary TB	Safety, immunogenicity, recurrence of TB disease	H56:IC31 was safe and immunogenic but did not reduce the recurrence of TB when compared to placebo.	[120]
11.	AERAS-402/AD35.TB-S (Ag85A + Ag85B + TB10.4)	Recombinant viral-vector vaccine	Phase II clinical trial	BCG-vaccinated infants	Safety and immunogenicity study, CD4^+^ and CD8^+^ immune responses	Vaccine was well tolerated and induced does-dependent cellular immune responses; no protective immunity was established.	[115]
12.	AERAS-402/AD35.TB-S (Ag85A + Ag85B + TB10.4)	Recombinant viral-vector vaccine	Clinical trial	HIV infected, BCG-vaccinated adults	Safety and immunogenicity study	Induced Ag85B-specific CD4^+^ and CD8^+^ T-cell responses in HIV-infected adults	[116]
13.	AERAS-422 (Ag85A + Ag85B + Rv3407)	Recombinant viral-vector vaccine	Phase I	Healthy adults (BCG-immunized naïve) First-in-human study	Safety and immunogenicity study	Strong immune response, but development halted due to safety concerns	[111]

### 3.9. From Immunogenicity to Protection: Unresolved Challenges

Though Ag85B-containing vaccines have repeatedly exhibited robust Ag-specific immune responses, the correlation between Ag85B immunogenicity and clinical protection remains incompletely defined. The H4 candidate, which incorporates Ag85B with TB10.4, is an important example: while demonstrating strong Ag-specific multifunctional CD4^+^ T-cell responses in BCG-vaccinated participants, vaccination did not significantly reduce the risk of Mtb infection in a phase II trial [121]. These observations highlighted that the extent of IFN-γ-production or antigen-specific T-cell responses alone may not represent a validated measure of protection [124,125]. Protective immunity against TB likely depends on numerous qualitative and structural attributes, including the balance between Th1 and Th17 immunity, the functionality of CD4^+^ and CD8^+^ T-cell responses, innate immune invasion, tissue-resident memory T cells in the lung, and mucosal response [125,126]. Notably, organized immune responses measured in peripheral blood may not adequately reflect the immune events occurring at the pulmonary site of Mtb infection. Immune cells appropriately localized and rapidly responsive at the site of infection may be critical for early infection control.

The gap between immunogenicity and protection may also reflect significant variation in both the host and the pathogen. Previously, BCG vaccination and pre-existing mycobacterial contact could shape vaccine-induced immunity, while differences in host immune background, vaccination route, antigen/adjuvant formulation, and Mtb strain may influence protective efficacy. Furthermore, changes in antigen expression during different stages of Mtb infection and the complex immunological environment associated with latent infection may limit the effectiveness of responses against individual antigens. Hence, although Ag85B remains a valuable and markedly immunogenic vaccine antigen, robust antigen-specific responses should be considered an essential prerequisite rather than a substitute for clinical protection. Subsequent vaccine development strategies may therefore benefit from combining Ag85B with complementary antigens and delivery or adjuvant methods that can generate more diverse, sustained, and targeted immune responses [121,125].

## 4. Conclusions and Future Perspective

Even after decades of research, the development of a highly effective vaccine against TB remains a significant global challenge. The most significant hurdle remains the limited understanding of robust immune markers for protection against TB infection. Numerous preclinical and clinical studies indicate that protection against TB is closely linked to antigen-specific T-cell responses, particularly CD4^+^ T cells producing IFN-γ, IL-2, and TNF-α [7,11,33,94,111,115]. In this regard, Ag85B has repeatedly emerged as an important immunodominant antigen that elicits a robust cellular immune response, leading to its widespread incorporation into multiple vaccine platforms engineered to enhance immunity beyond traditional BCG.

Although Ag85B-based vaccines have shown encouraging results in both preclinical and clinical trials, translating them into long-lasting, consistent protection remains a critical challenge. Emerging evidence indicates that an effective TB vaccine may require coordinated protection from both adaptive and innate immune responses. Innate immunity-associated processes, such as inflammasome activation and autophagy, play a key role in shaping host response and protection against mycobacterial infection [74,127,128]. Therefore, incorporating these pathways into vaccine design through enhanced delivery systems, targeted adjuvant selection, and advanced genetic engineering may increase the efficacy of Ag85B-based vaccines. Another potential approach involves enhancing antigen presentation and immune targeting via advanced vaccine platforms. New strategies, including nanoparticle-based delivery systems, viral vectors, and recombinant bacterial vectors, offer new possibilities to enhance the immunological potential of Ag85B [94,110]. These approaches help increase cellular uptake and improve antigen stability, leading to enhanced T cell activation. The incorporation of advanced technologies, such as proteomics and transcriptomics, into vaccine development may help uncover novel aspects of immune regulation during TB infection [94].

Beyond vaccine development, recent studies have highlighted Ag85B as a valuable protein of interest for wide-ranging translational applications. It is being explored in diagnostic analyses, immune monitoring studies, and host–pathogen interaction studies during TB infection [129,130,131,132,133]. Moving ahead, future research should seek to incorporate these novel insights into a more integrated framework for TB control. Fusion of Ag85B with other immunogenic antigens and a deep understanding of its mechanism in generating integrated innate and adaptive immune responses may guide the development of the next-generation TB vaccine. Beyond being considered a vaccine agent, Ag85B is now considered a multifunctional research target with potential applications in TB theranostics. Leveraging this broader potential may ultimately facilitate the development of efficient strategies for the diagnostics, prevention, and control of TB.

## Data Availability

No new data were created or analyzed in this study. Data sharing is not applicable to this article.
